# The Characteristics of Patients Associated With High Caregiver Burden in Parkinson's Disease in Singapore

**DOI:** 10.3389/fneur.2019.00561

**Published:** 2019-05-28

**Authors:** Mark M. J. Tan, Ee Chien Lim, Nivedita Vikas Nadkarni, Weng Kit Lye, Eng King Tan, Kumar M. Prakash

**Affiliations:** ^1^Duke-NUS Medical School, Singapore, Singapore; ^2^Department of Neurology, National Neuroscience Institute, Singapore, Singapore; ^3^Centre for Quantitative Medicine, Duke-National University of Singapore (NUS) Medical School, Singapore, Singapore

**Keywords:** Parkinson's disease, levodopa motor complication, non-motor symptoms, quality of life, caregiver burden

## Abstract

**Introduction:** Given the complex multitude of Parkinson's disease (PD) symptoms, caregiving for PD patients can be highly demanding. Our study was aimed to investigate the characteristics of PD patients related to different levels of caregiver burden.

**Methods:** This cross-sectional study recruited 104 idiopathic PD patient-caregiver pairs. Patients were evaluated on motor, non-motor symptoms, and quality of life (QoL). Caregiver burden was quantified using Zarit Burden Inventory and subsequently stratified into 3 subgroups. Statistical analysis was performed to identify differences in the no-or little, mild-moderate, and high caregiver burden subgroups.

**Results:** The mean disease duration was significantly longer in the high caregiver burden group compared to no-or little group (9.63 vs. 5.17 years; *p*-value 0.003). The mean levodopa equivalent daily dose (LEDD) and mean total UPDRS Part IV scores (UPDRS4) were significantly higher in the high caregiver burden group compared to no-or little group (*p*-value 0.011 and 0.004, respectively). The high caregiver burden group had significantly higher median QoL scores (PDQ-39) for PD patients for domain 2 (ADL, *p*-value 0.005), domain 4 (stigma, *p*-value 0.005), and domain 6 (cognition, *p*-value 0.002) compared to no-or little group.

**Conclusion:** Greater caregiver burden was observed in PD patients with more prolonged disease duration, higher LEDD to control motor symptoms as well as greater levodopa related motor complications. Further studies on potential interventions to mitigate or delay levodopa related motor complications may reduce caregiver burden. Marked worsening in patient's QoL, specifically ADL, stigma and cognition in the high compared to no-or little caregiver burden group suggests the possible utility of monitoring these factors for early identification of increasing caregiver stress and burden.

## Introduction

Parkinson's disease (PD) is a neurodegenerative disorder causing progressive motor and non-motor symptoms (1). Motor symptoms, in particular gait impairment and long-term motor complications arising from levodopa therapy, have been shown to significantly affect the health-related quality of life (QoL) (1). Similarly the non-motor symptoms (NMS) in PD patients have also significantly contributed to a lower health-related QoL (2). These NMS include cognitive impairment, sleep disturbances and mood-related issues. In addition, individuals with more advanced stages of disease and higher levels of disability experienced poorer QoL (2–4). These findings were reproduced in our longitudinal studies as well. In fact, we found that the non-motor problems provided a better prediction for the change in QoL compared to motor symptoms (5).

PD symptoms not only affect the patient‘s QoL but also have an impact on the burden and QoL of their caregivers (1). With the progressive nature of the disease, demands on the caregivers are likely to increase in the later stages when patients become more physically dependent for their activities of daily living (6–8). A recent study in Spain suggested that PD duration, disease stage, depressive symptoms, behavioral, and sleep disturbances as well as dementia were found to be associated caregivers' stress and burden (1). However, these observations and findings are likely less generalizable due to the differences in cultural contexts internationally. For example, in Asian societies with oriental origin, where Confucianism ideals have a large influence on filial piety and family dynamics (6, 7), it is not surprising that families opt to take on the burden of care for their affected family members at home. Notably, in Singapore, there exists a spectrum of varying family dynamics (7, 8) where care for the patient may fall on an elderly spouse, children or even hired live-in helpers, leading to possibly more complex caregiver burden.

Till date, little is known with regards to caregiving for PD patients locally and the long term consequences on the caregivers. There is only a qualitative report on caregivers' experiences in caring for local PD patients; no quantitative analysis with regards to factors related to high caregiver burden has been assessed (9).

Therefore, the primary objective of this study was to evaluate the characteristics of the patients with PD associated with different levels of caregiver burden. These factors may then be further investigated for the necessary early intervention for better patient and caregiver outcomes.

## Methods

### Recruitment and Criteria

This was a cross-sectional observational study. A total of 104 idiopathic PD patients, diagnosed according to the UK PD Brain Bank criteria (10), and their caregivers were prospectively recruited from the movement disorders clinics in a tertiary referral center in Singapore (between 2015 and 2017). Written informed consent was obtained from all participants of the study.

For this study, a caregiver was defined as an unpaid primary caregiver of the care recipient; who provided a minimum of 3 h of care per day for a minimum of 6 months; and was older than 21 years of age and able to give consent.

Patients with a diagnosis of Parkinsonism secondary to alternative causes were excluded. Those with severe debilitating conditions such as severe heart failure or other terminal illness were also excluded.

The study was approved by the Singhealth Centralized Institutional Review Board and performed in accordance to relevant guidelines and regulations.

### Assessments

All patients were evaluated by neurologists and trained research associates. If the interview could not be completed within one session for any reason, follow-up telephone interviews were conducted within 2 weeks. All patients underwent evaluations and examinations involving the following assessment measures:

Socio-demographic data including age, gender, ethnicity, marital status, level of education, and occupation, were obtained from all recruited patients.

The primary outcome, PD patient's caregiver burden, was assessed with the Zarit Burden Inventory (ZBI) (11). ZBI has been specially designed to reflect the stresses experienced by caregivers of patients. Caregivers were asked to respond to a series of 22 questions about the impact of the patient's disabilities on their life. For each item, caregivers had to indicate how often they felt that way (never, rarely, sometimes, quite frequently, or nearly always). In addition, patients or their family members were asked if a hired live-in helper was also involved in the care of the PD patients.

NMS were assessed using the Non-Motor Symptoms Scale (NMSS) (12, 13), a validated scale that evaluates non-motor symptoms experienced over the past month. The NMS are grouped into nine domains (cardiovascular, sleep and fatigue, mood, and apathy, perceptual problems and hallucination, attention and memory, gastrointestinal, urinary, sexual function, and miscellaneous). Each item in the NMSS is rated based on severity (scored from 0 to 3) and frequency (scored from 1 to 4). The frequency score is then multiplied with the severity score to give an NMS burden score. A domain score is calculated from the total of all NMS burden within a domain, and a total NMS burden score is derived as the sum of all domain scores.

PD patient's QoL was assessed using a Parkinson's disease-specific instrument, the Parkinson's Disease Questionnaire-39 (PDQ-39) (9). It comprises of 39 items grouped into eight domains: mobility, activities of daily living, mood/emotional well-being, stigma, social support, cognition, communication, and bodily discomfort. Responses are scored from a scale of 0 (never) to 4 (always). Domain scores are summed from each item's raw score and divided by the maximum possible raw score of the domain and converted to a percentage. A Summary Index (PDQSI) is then calculated as the average of all eight domain scores weighted evenly by domain. Higher scores reflect a poorer QoL.

Parkinson's disease patients were assessed for motor disabilities during the OFF-state using Part III (motor) Unified Parkinson's Disease Rating Scale (UPDRSm) (10) and the modified Hoehn and Yahr (H&Y) staging scale (14). Complication of therapy was assessed using UPDRS Part IV scores (UPDRS4) (15). The ability to perform daily activities was assessed using the Schwab and England Activities of Daily Living (ADL) score (16). The levodopa equivalent daily dose (LEDD) was calculated according to standardized formulae: ([levodopa (mg)] + [controlled release levodopa (mg)^*^0.75] + [levodopa doses taken together with entacapone (mg)^*^1.33] + [pramipexole (mg)^*^100] + [ropinirole (mg)^*^20] + [piribedil (mg)^*^1] + [bromocriptine (mg)^*^10] + [rotigotine (mg)^*^30]) (17).

The above assessments were carried out face-to-face with patients on the same days as their specialist clinic visit. All patients carried on receiving symptomatic therapy (either pharmacological or non-pharmacological therapy or in most cases both) if deemed necessary by the treating specialist.

### Statistical Analysis

The Shapiro-Wilk test of normality was performed on the variables to evaluate the assumption of normality. Continuous data were summarized as mean (standard deviation) or median (interquartile range) for symmetrically distributed and skewed data, respectively. Categorical data were summarized by frequency (%).

Patients were subdivided into 3 groups based on their ZBI scores: 0–20 for no-or little burden, 21–40 for mild-moderate burden, and >40 for high burden. This stratification was to evaluate the likelihood of factors associated with different levels of caregiver burden. One-way ANOVA or the Kruskal-Wallis test based on symmetrically distributed and skewed data, respectively, was performed to test for differences in the demographics, UPDRSm, UPDRS4, ADL, NMSS and PDQ-39 scores among the 3 groups. The Chi-Square test was performed between groups to test for association between the categorical ZBI and H&Y staging (up to stage 2 or more than stage 2), gender, education level (up to secondary or above secondary level) and the presence or absence of domestic helper(s).

Where the multiple comparisons across the three groups were found to be significantly different (*p* < 0.05), we performed a *post-hoc* pairwise testing (with *p* < 0.0167 (0.05/3) considered as statistically significant). For those variables, which had the ANOVA to test three way comparisons, Tukey's *post-hoc* pairwise test was performed. For those variables, which had the Kruskal Wallis to test the three way comparisons, Dunn's *post-hoc* pairwise test was performed. All analyses were performed in SPSS (Version 24).

## Results

### Demographics

A majority of the patients were males (64.4%), of Chinese ethnicity (91.3%), retirees (91.3%), educated up to secondary level (81.7%), and were in early stages of PD with a H&Y staging under 3 (71.2%) (Table 1). Based on the ZBI scores, 47 reported no-or little caregiver burden, 41 reported mild-moderate caregiver burden, while 16 reported high caregiver burden.

**Table 1:** Characteristics of the PD patients (*n* = 104).

**Characteristics**	**Mean (SD) or Median (IQR) or Frequency (%)**
**Patient**	
Age	68.9 (8.40)
Gender (Male)	67 (64.4%)
Race (Chinese)	95 (91.3%)
Education (higher than secondary)	19 (18.3%)
Occupation (Retiree)	95 (91.3%)
Disease duration (Years)	6.23 (4.70)
Hoehn and Yahr (Stage 3 and above)	30 (28.8%)
Total NMS burden	31.50 (16.00,49.75)
Total UPDRSm	26.8 (14.5)
Schwab & England ADL score	82.0% (15.8)
PDQSI	21.1% (10.1,30.8)
ZBI Score	24.6 (15.3)
0–20	47 (45.2%)
21–40	41 (39.4%)
>40	16 (15.4%)

*ADL, Activities of Daily Living; IQR, interquartile range; LEDD, levodopa equivalent daily dose; NMS, non-motor symptoms; UPDRSm, Unified Parkinson's Disease Rating Scale Part III (motor); PDQSI, Parkinson's Disease Questionnaire Summary Index; ZBI, Zarit Burden Inventory*.

### Disease Duration, LEDD, UPDRSm and UPDRS4

The patients were stratified according to their caregiver burden to compare various characteristics between groups using the ANOVA testing (Table 2).

**Table 2:** ANOVA test on patients' characteristics grouped by caregiver burden severity.

	**No-or little (ZBI** **=** **0–20)** ***n*** **=** **47**		**Mild-moderate (ZBI** **=** **21–40)** ***n*** **=** **41**		**High (ZBI** **>** **40)** ***n*** **=** **16**		
	**Mean**	**SD**	**Mean**	**SD**	**Mean**	**SD**	**ANOVA *p*-value**
Age (Years)	68.98	8.71	68.88	8.41	69.00	7.96	0.998
Disease duration (Years)	5.17	4.29	6.12	4.28	9.63	5.54	0.004
UPDRSm (Total)	25.98	14.62	26.05	13.88	31.19	15.91	0.427
UPDRSm (Speech)	0.87	0.70	1.17	0.80	1.47	0.99	0.025
UPDRSm (Bradykinesia)	11.20	6.68	11.96	6.40	15.19	8.28	0.135
UPDRSm (Rigidity)	5.09	3.42	5.39	3.12	6.84	4.31	0.213
UPDRSm (Tremor)	3.45	4.21	2.17	2.57	1.38	2.03	0.058
UPDRSm (Postural Stability & Gait)	4.09	2.62	5.18	2.94	6.31	3.65	0.024
UPDRS4 (Total)	2.13	2.44	2.66	2.17	4.44	2.78	0.005
UPDRS4 (Dyskinesia)	0.45	1.18	0.39	1.05	1.31	1.85	0.036
UPDRS4 (“OFF” symptoms)	1.28	1.31	1.73	1.40	1.88	1.26	0.166
LEDD	299.7	235.8	397.3	295.6	556.4	317.2	0.013
ADL	0.86	0.16	0.80	0.15	0.75	0.13	0.035
ECAQ	8.64	2.14	7.90	2.61	7.88	2.00	0.270

*ADL, Schwab and England Activities of Daily Living; ECAQ, Elderly Cognitive Assessment Questionnaire; LEDD, levodopa equivalent daily dose; NMSs, non-motor symptoms; UPDRSm, Unified Parkinson's Disease Rating Scale Part III (motor); UPDRS4, Unified Parkinson's Disease Rating Scale Part IV; PDQSI, Parkinson's Disease Questionnaire Summary Index; ZBI, Zarit Burden Inventory; SD, Standard Deviation*.

The mean age and ECAQ scores did not show statistically significant differences among the groups. Traversing from no-or little, mild-moderate to high caregiver burden groups, there were significant difference in the mean disease duration (5.17 to 6.12 to 9.63 years, *p*-value 0.004). *Post-hoc* test showed that the mean disease duration was significantly longer in the high caregiver burden group compared to no-or little group (*p*-value 0.003). The mean LEDD was significantly higher in the high caregiver burden group compared to no-or little group (556mg vs. 300mg, *p*-value 0.011). The mean ADL score was lower (although not statistically significant) in the high caregiver burden group compared to no-or little group (75% vs. 86%, *p*-value 0.047).

There was no significant differences between the groups for mean total UPDRSm scores after adjusting for pairwise comparisons using the Bonferroni correction. However, the mean total UPDRS4 score was significantly higher in the high caregiver burden group compared to no-or little group (*p*-value 0.004).

### Gender, Education Level, H&Y Stage and Live-in-Helper

The gender and education level distribution across the groups was consistent with the overall demographics and did not show statistically significant differences among the groups (*p*-value 0.822 and 0.183, respectively). The proportion of patients with H&Y stage 3 and above increased across the ZBI categories (12.8 to 34.1 to 56.3%) however the difference was not statistically significant (*p*-value 0.140). There was also a marked increment in the hiring of live-in helpers across the caregiver burden groups (17.0 to 14.7 to 43.8%) but the association was not statistically significant (*p*-value 0.053).

### NMSS and PDQ

The patients were similarly stratified according to their caregiver burden to compare the NMSS and PDQ scores between groups using the Kruskal-Wallis test (Tables 3,4).

**Table 3:** Kruskal-Wallis test on patients' NMSS scores grouped by caregiver burden severity.

	**No-or little (ZBI = 0–20) *n* = 47**	**Mild-moderate (ZBI = 21–40) *n* = 41**	**High (ZBI > 40) *n* = 16**	
	**Median (IQR)**	**Median (IQR)**	**Median (IQR)**	***p*****-value**
NMSS Total	26.0 (16.0, 46.0)	31.0 (17.0, 45.0)	48.0 (32.0, 58.8)	0.027
Domain 1 *Cardiovascular*	0.0 (0.0, 1.0)	0.0 (0.0, 1.0)	0.0 (0.0, 3.5)	0.461
Domain 2 *Sleep/Fatigue*	5.0 (0.0, 8.0)	8.0 (2.0, 12.5)	9.0 (4.5, 18.3)	0.044
Domain 3 *Mood/Apathy*	3.0 (0.0, 8.0)	1.0 (0.0, 8.0)	8.0 (4.0, 12.8)	0.009
Domain 4 *Perception*	0.0 (0.0, 0.0)	0.0 (0.0, 1.0)	0.0 (0.0, 4.0)	0.002
Domain 5 *Attention*	2.0 (0.0, 4.0)	2.0 (0.0, 8.0)	5.0 (0.5, 12.0)	0.119
Domain 6 *Gastrointestinal*	1.0 (0.0, 4.0)	4.0 (0.0, 8.0)	2.5 (0.5, 10.0)	0.253
Domain 7 *Urinary*	5.0 (1.0, 12.0)	4.0 (0.0, 9.0)	6.0 (2.5,8.8)	0.431
Domain 8 *Sexual*	0.0 (0.0, 0.0)	0.0 (0.0, 0.0)	0.0 (0.0, 0.0)	0.340
Domain 9 *Miscellaneous*	2.0 (0.0, 6.0)	1.0 (0.0, 5.5)	2.0 (0.3, 4.0)	0.671

*NMSs, non-motor symptoms; ZBI, Zarit Burden Inventory; IQR, interquartile ranges*.

**Table 4:** Kruskal-Wallis test on patients' PDQ-39 scores grouped by caregiver burden severity.

	**No-or little (ZBI = 0–20) *n* = 47**	**Mild-moderate (ZBI = 21–40) *n* = 41**	**High (ZBI > 40) *n* = 16**	
	**Median (IQR)**	**Median (IQR)**	**Median (IQR)**	***p*****-value**
PDQSI	16.2 (8.6, 23.5)	23.2 (10.6, 31.7)	30.7 (23.0, 48.9)	0.002
Domain 1 *Mobility*	22.5 (5, 37.5)	40.0 (16.3, 68.8)	50.0 (18.1, 83.8)	0.015
Domain 2 *ADL*	8.3 (0, 16.7)	25.0 (2.08, 50)	37.5 (9. 4, 71.9)	0.005
Domain 3 *Mood*	12.5 (0, 25.0)	16.7 (2.08, 33.3)	35.4 (11.5, 59.4)	0.014
Domain 4 *Stigma*	0.0 (0, 18.8)	0.0 (0, 12.5)	31.3 (1.6, 56.3)	0.005
Domain 5 *Social support*	0.0 (0, 8.3)	0.0 (0, 4.2)	0.0 (0, 29.2)	0.691
Domain 6 *Cognition*	18.8 (6.3, 31.3)	25.0 (12.5, 43.8)	43.8 (26.6, 60.9)	0.002
Domain 7 *Communication*	8.3 (0, 33.3)	0.0 (0, 33.3)	33.3 (12.5, 62.5)	0.021
Domain 8 *Body discomfort*	16.7 (0, 33.3)	16.7 (0, 37.5)	33.3 (4.2, 58.3)	0.153

*PDQSI, Parkinson's Disease Questionnaire Summary Index; ZBI, Zarit Burden Inventory; IQR, interquartile ranges*.

The median total NMSS score was higher (but not statistically significant) in the high caregiver burden group compared to no-or little group (48.0 vs. 26.0, *p*-value 0.023). Out of the 9 domains, only domains 3 (Mood and Apathy) and 4 (Perception and hallucinations) showed statistically significant difference in the median scores of the 3 groups (Table 3). However, after the *post-hoc* test, adjusting for pairwise comparisons using the Bonferroni correction, there was no significant differences between 2 caregiver burden groups for all the domains.

The median PDQSI scores was significantly higher in the high caregiver burden group compared to no-or little group (30.7 vs. 16.2, *p*-value 0.002). Out of the 8 domains of PDQ, domains 5 (social support), 7 (communication), and 8 (bodily discomfort) did not show statistically significant difference in the median scores among the 3 groups (Table 4). After the *post-hoc* test, adjusting for pairwise comparisons using the Bonferroni correction, the high caregiver burden group had significantly higher median scores for domain 2 (ADL, *p*-value 0.005), domain 4 (stigma, *p*-value 0.005), and domain 6 (cognition, *p*-value 0.002) compared to no-or little group.

## Discussion

The causes of caregiver burden in PD are multifactorial. Previous studies from the western part of the globe have established that motor and non-motor symptoms affect patients and caregivers on a day-to-day basis (1). This study attempted to investigate on PD patient factors associated with different levels of caregiver burden in Singapore.

### Disease Duration, LEDD and UPDRS4

In our study, PD patients with a longer duration of disease and on higher LEDD were associated with higher caregiver burden. Previous studies have also shown strong correlation between caregiver burden and disease duration (18, 19). In addition, our study also showed that patients with higher UPDRS4 scores were also associated with high caregiver burden. This observation is not surprising as motor fluctuation and dyskinesia are commonly seen in patients with longer disease duration and higher LEDD as a result of the narrow therapeutic window of levodopa therapy in advanced stage PD (20).

However, it was interesting to note that the UPDRSm score (severity of motor symptoms) did not show significant difference among the various caregiver burden groups. Similarly, although the proportion of patients with higher H&Y stage increased across the caregiver burden groups, the difference was not statistically significant. This may suggest that the classic motor symptoms (rigidity, bradykinesia, tremor, and postural instability) and disease staging related to these motor symptoms which are assessed by physicians or trained raters may not significantly influence the severity of caregiver burden as compared to the subjective reporting by patients about the limitations due to levodopa therapy related complications.

### Dependency on Caregiver

The median PDQ-39 domain scores for ADL limitations showed a significant worsening from the no-or little to high caregiver burden groups. While it may be intuitive that decline in the ADL would translate to higher dependency on caregivers, the findings also suggest that patient's self-reporting of ADL limitations have impact on caregiver burden and this could be a window of opportunity to intervene and reduce or delay the caregiver strain.

### Stigma and Cognition

Multiple studies (1, 18, 19, 21) have demonstrated that neuropsychological symptoms are the strongest predictor for poor patient health related QoL and have been shown to increase caregiver burden in PD and dementia (15). In our study, social stigma (PDQ-39 domain 4) and cognitive problems (PDQ-39 domain 6) were shown to be more prevalent in the high caregiver burden group compared the no-or little group. In general, cognitive and psychological symptoms are often overshadowed by the motor symptoms and are under-recognized in PD (22). Furthermore in Asian societies, these symptoms are often stigmatized and therefore are under-reported, under-diagnosed and under-treated (23). In view of such limitations, future studies should be aimed to investigate the incidence of PD patients with significant neurobehavioral symptoms and the impact of treating these symptoms on patient related outcome and caregiver burden.

### Live-in Helpers

There was an increment in the hiring of live-in helpers across the caregiver burden groups (17.0 to 14.7 to 43.8%), however the difference was not statistically significant. Within the high caregiver burden group, although a majority had indicated their spouse as the main caregiver, almost half of them also reported their live-in helper as a caregiver, some of whom as the main caregiver.

In Singapore, it is also not uncommon for families of multi-generation living together in the same household and the care burden of a patient may be distributed among several members of the household. Therefore, the stress on them as well as the hired help often goes unrecognized (24). In view of these culture-specific dynamics, future studies could explore on the burden of care and stress on more members within the household.

### Limitations

The study sample size is not robust, however, we believe the results maybe of relevance for future studies. In addition although the data suggests that disease duration may be associated with increasing caregiver burden, it is not possible to evaluate a temporal relationship from a cross-sectional study. A longitudinal study would be more appropriate to examine transitions between different degrees of caregiver burden, and would ascertain if factors seen in early disease may indeed herald evolving caregiver burden. The progression time would provide further insights on the window of opportunity for intervention. For this study, the patient-caregiver pair had to possess sufficient cognitive ability and give consent to participate. Exclusion of those who did not meet the criteria may have resulted in omission of possible high caregiver burden carers. However, we believe that the patient-caregiver PD population in our study would still be relevant to understand the caregiver burden in an Asian sociocultural context and explore opportunities for timely intervention. Lastly the socioeconomic status of patients was not evaluated which perhaps should be investigated in future studies.

## Conclusion

Our study showed that groups with greater caregiver burden were more likely to consist of patients with more prolonged disease duration, higher LEDD to control motor symptoms as well as greater levodopa related motor complications. Further studies on potential interventions to mitigate or delay levodopa related motor complication in PD patients may reduce caregiver burden.

Marked worsening in patient's QoL domains, specifically ADL, stigma and cognition in the high compared to no-or little caregiver burden group suggests the possible utility of monitoring these factors for early identification of increasing caregiver stress and burden. Early identification and intervention may decrease caregiver burden and burnout and ultimately translate to better outcomes for both patients and caregivers.

## Ethics Statement

The study was approved by the SingHealth Centralized Institutional Review Board and performed in accordance to relevant guidelines and regulations.

## Author Contributions

MT, KP, and ET were involved in the conception of the work. MT, EL, KP, and ET were involved with the recruitment of patients and collection of data. MT, NN, WL, and KP were involved in data analysis and interpretation. MT and KP drafted the manuscript. All authors reviewed the manuscript.

### Conflict of Interest Statement

The authors declare that the research was conducted in the absence of any commercial or financial relationships that could be construed as a potential conflict of interest.
